# Profilin 2 and Endothelial Exosomal Profilin 2 Promote Angiogenesis and Myocardial Infarction Repair in Mice

**DOI:** 10.3389/fcvm.2022.781753

**Published:** 2022-04-11

**Authors:** Zhenkun Li, Xueyun Huo, Keyan Chen, Fenghua Yang, Weijiang Tan, Qi Zhang, Haixu Yu, Changlong Li, Deshan Zhou, Hao Chen, Baoquan Zhao, Yuan Wang, Zhenwen Chen, Xiaoyan Du

**Affiliations:** ^1^School of Basic Medical Sciences, Capital Medical University, Beijing Key Laboratory of Cancer Invasion & Metastasis Research, Beijing, China; ^2^Experimental Center, Beijing Friendship Hospital, Capital Medical University, Beijing, China; ^3^Department of Laboratory Animal Science, China Medical University, Dalian, China; ^4^Guangdong Laboratory Animals Monitoring Institute, Guangdong Provincial Key Laboratory of Laboratory Animals, Guangzhou, China; ^5^Beijing Anzhen Hospital, Capital Medical University, Beijing, China; ^6^State Key Laboratory of Toxicology and Medical Countermeasures, Institute of Pharmacology and Toxicology, Beijing, China

**Keywords:** angiogenesis, endothelial cells, exosomes, myocardial infarction, profilin 2

## Abstract

Cardiovascular diseases (CVD) are the leading cause of death worldwide, wherein myocardial infarction (MI) is the most dangerous one. Promoting angiogenesis is a prospective strategy to alleviate MI. Our previous study indicated that profilin 2 (PFN2) may be a novel target associated with angiogenesis. Further results showed higher levels of serum PFN2 and exosomal PFN2 in patients, mice, and pigs with MI. In this study, we explored whether PFN2 and endothelial cell (EC)-derived exosomal PFN2 could increase angiogenesis and be beneficial for the treatment of MI. Serum PFN2, exosomes, and exosomal PFN2 were elevated in rats with MI. PFN2 and exosomes from PFN2-overexpressing ECs (OE-exo) enhanced EC proliferation, migration, and tube formation ability. OE-exo also significantly increased the vessel number in zebrafish and protected the ECs from inflammatory injury. Moreover, OE-exo-treated mice with MI showed improvement in motor ability, ejection fraction, left ventricular shortening fraction, and left ventricular mass, as well as increased vessel numbers in the MI location, and decreased infarction volume. Mechanistically, PI3K might be the upstream regulator of PFN2, while ERK might be the downstream regulator in the PI3K-PFN2-ERK axis. Taken together, our findings demonstrate that PFN2 and exosomal PFN2 promote EC proliferation, migration, and tube formation through the PI3K-PFN2-ERK axis. Exosomal PFN2 may be a valuable target in the repair of MI injury via angiogenesis.

**Graphical Abstract:**
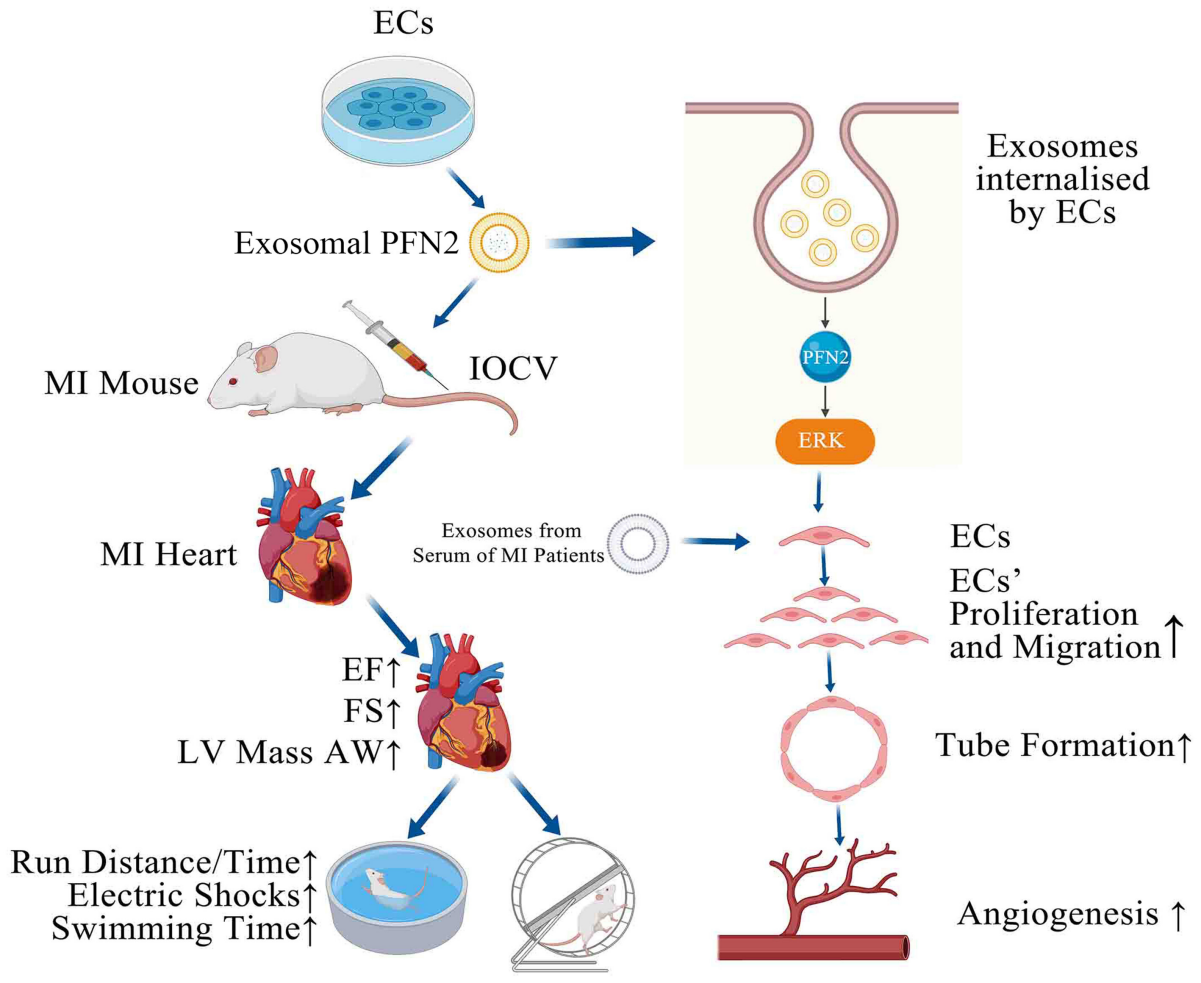
PFN2-overexpressing ECs (OE-exo) treated MI mice showed improvement in infarction volume, cardiac function and motor ability, and PFN2/ OE-exo significantly enhanced EC proliferation, migration, tube formation ability and angiogenesis.

## Introduction

Myocardial infarction (MI) is caused by ischemia, a condition referring lack of oxygen delivery to heart tissues. Ischemic heart disease is responsible for over 9 million deaths in 2016 according to the World Health Organization estimates (1). MI leads to cardiac myocyte death and subsequent necrosis of the tissue in the infarcted area, attracting inflammatory cells that phagocytose dead cells and debris within the infarcted area (2). Infarct size is a major indicator of post-MI remodeling, subsequent heart failure (3), and eventually prognosis (4–6), and in the longer term, inflammation contributes to changes associated with an increased likelihood of heart failure and mortality (7, 8). During MI, cardiomyocytes die as a result of transmural ischemia (9). One of the most important biological processes during myocardial healing after ischemic injury is microvascular angiogenesis, which consists of the development of new blood vessels from pre-existing vasculature (10). Angiogenesis is stimulated by increased levels of vascular endothelial growth factor (VEGF) and basic fibroblast growth factor (bFGF), which are released in response to the infarction. Recent study has shown that the promotion of angiogenesis in the ischemic region is the promising therapeutic strategies for preventing MI (11). Researchers found that irisin exerts a therapeutic effect against myocardial infarction via promoting angiogenesis (12) and a novel Ca^2+^ current blocker promotes angiogenesis and cardiac healing after experimental myocardial infarction in mice (13). Therefore, it is particularly important to explore new molecules and therapy pathways that relieving the adverse effects of inflammation and promoting angiogenesis after myocardial infarction.

Exosomes can deliver a wide range of functional molecules such as proteins, RNAs, and genomic DNA (14–16). Evidence has shown that exosomes deliver bioactive molecules to endothelial cells (ECs) to induce angiogenesis (17), and ECs could also release exosomes to mediate the formation of blood vessels (18). Recent research has highlighted the significant potential of pericardial fluid- and plasma-derived exosomes as therapeutic angiogenesis targets in patients with MI (19, 20). Although exosomes have been shown to play an important role in angiogenesis (21), our knowledge of cardiac repair medicated by vascular EC-derived exosomes post MI is very limited.

Profilin (PFN), a 12–15 kDa actin-binding protein, is ubiquitously expressed and highly conservative (22). PFNs participate in many cellular processes such as cell motility, metabolism, signal transduction and gene transcription (23). Among PFN Isoforms, profilin 2 (PFN2) has been known to play key roles in tumor progression. Zhang H et al. found loss of PFN2 contributes to enhanced epithelial-mesenchymal transition and metastasis of colorectal cancer (24) and Tang et al. reported that PFN2 promoting TGF-β-mediated EMT and increased VEGF expression (25). But the effects of PFN2 on the function of endothelial cells and inflammation after MI have been unknown. Most recently, we found that PFN2 and exosomal PFN2 could promote small cell lung cancer growth and metastasis as well as tumor angiogenesis (26), we speculated that exosomal PFN2 might also promote angiogenesis on cardiac tissue after MI.

In the present study, we hypothesized that MI release exosomes carrying PFN2, which promotes angiogenesis. Therefore, we examined the serum of patients with MI and found that levels PFN2, exosomes, and exosomal PFN2 were increased during angiogenesis phase. We further demonstrated that the beneficial function of PFN2 and endothelial exosomal PFN2 in the proliferation, migration, and tube formation of ECs. Moreover, exosomal PFN2 derived from ECs has a significant therapeutic effect in mice with myocardial infarction. We also revealed that PFN2 were involved in PI3K -PFN2-ERK axis during angiogenesis.

## Materials and Methods

### MI Patients

According to the period from symptom onset to diagnosis, we divided the samples into 2–10 h of MI group (*n* = 30), 8–15 days after MI group (*n* = 30), and healthy controls group (*n* = 24). All patients provided informed consent and the patient information is listed in Supplementary Table 1.

#### Inclusion Criteria

(1) Meet the diagnostic criteria of acute myocardial infarction;
(2) Patients with unconscious disorder can actively cooperate with medical staff;
(3) Patients who have informed the purpose, method and significance of the study, voluntarily participated in the study and signed the informed consent.

#### Exclusion Criteria

(1) Complicated with serious organ diseases;
(2) Incomplete clinical data.

### Myocardial Infarction Models of Mice, Rats and Pigs

A total of 15 male Wistar rats (230–280 g, 7–8 weeks old) were utilized in the study. The animals were maintained under controlled conditions with a temperature of 24 ± 2°C, humidity of 55–65%, a 12 h light/dark cycle, and *ad libitum* access to standard laboratory diet and water.

#### Rodent MI Models

Briefly, surgery was performed under isoflurane (2.5%) inhalation anesthesia and mechanical ventilation. The MI model was established by performing a left lateral thoracotomy and ligating the left anterior descending artery (LAD) using a 10-0 suture. The sera were collected before and after surgery at different time points to evaluate the expression of PFN2, exosomes, and exosomal PFN2.

The experimental pigs were anesthetized with ketamine and maintained with isoflurane. kept in the right lateral position, cut open the left rib 3–4, exposed the main coronary artery of the heart, ligated between the second and third branches, and the chest wall was sutured layer by layer after successful myocardial infarction confirmed by electrocardiogram. Serum samples from pigs with MI (*n* = 4) and their corresponding controls (*n* = 4) were collected at different time points after MI.

### Reagents

RPMI-1640 medium and phosphate-buffered saline (PBS) were purchased from Hyclone (USA), and Endothelial Cell Medium was from ScienCell (USA). MTT and DMSO were purchased from Beyotime (China). PI3K inhibitor LY-294002 and ERK1/2 inhibitor HY-50846 were from MCE (USA). The various antibodies used in the experiments were obtained from Abcam (UK).

### Isolation of Exosomes

Exosomes were isolated by the salting-out method (Serum) and ultracentrifugation (Cells culture medium with exosome-depleted fetal bovine serum). The serum sample was centrifuged at 2,000 × *g* for 20 min at room temperature to remove cells and debris. The supernatant was transferred gently to a new tube and centrifuged again at 10,000 × *g* for 20 min at room temperature to remove debris. The clarified serum was transferred to a new tube and 0.5 volumes of PBS and 0.3 volumes of exosomes isolation reagent were added. The sample was vortexed and centrifuged at 10,000 × *g* for 5 min at room temperature, and the supernatant was aspirated and discarded. Finally, the tube was centrifuged for 30 s at 10,000 × *g*, and the residual supernatant was discarded by careful aspiration. PBS was added, and the exosomes were resuspended by vortex. The cell culture medium was centrifuged at 300 × *g* for 10 min, and the supernatant was centrifuged at 2,000 × *g* for 10 min/ 10,000 × *g* for 30 min/140,000 × *g* for 90 min, PBS was added to the pellet, and the exosomes were resuspended by vortex. Exosomes were identified by transmission electron microscopy (TEM). Exosomes were stored at 4°C within a week or −80°C within 3 months. In cell co-culture experiences, the final concentration of exosomes was 10 μg/ml. In animal experiences, mice were injected with 50 μg exosomes mixed in 100 μl PBS.

### Immunofluorescence and Masson's Staining

After functional analysis, 21 mice with MI, with or without exosomes were euthanized, and the heart tissues were fixed in formalin and embedded in paraffin. Immunofluorescence analysis was performed on 4 μm thick myocardial tissue sections. The sections were incubated with primary antibodies against PFN2 (Abcam, ab191054, UK) or CD31^+^ (Abcam, ab24590, UK). Alexa Fluor 488- or Alexa Fluor 555-conjugated secondary antibodies (Invitrogen) were added and incubated for 1 h. After staining with Hoechst 33342 (Sigma-Aldrich) and sealing with an anti-fade fluorescence mounting medium (Solarbio), the images were captured.

For Masson's staining, the samples were treated with hematoxylin for 5 min, with deionized water for 30 s three times, and with Masson's dye (HT15, Sigma, USA) for 7 min. Then, the sections were treated with 2% glacial acetic acid solution and 10% molybdic acid for 5 min. After aniline blue staining for 5 min and gradient alcohol washing, neutral gum was used to seal the slide, and the sample was observed under a microscope.

### Exercise Endurance Capacity

A mouse model of MI was established using 21 mice with 7 animals in each group. The experimental procedure was similar to that of the rat model of MI. On the 5th day after MI, the animals were randomly divided into three groups and injected in the tail vein with profilin 2 overexpressing EC-exosomes (OE-exo), normal EC-exosomes (exo), and saline. After 21 days of injection, all animals were subjected to physical fitness (YLS-10B) and swimming endurance tests. Cardiac function was evaluated for ejection fraction (EF), fractional shortening (FS), left ventricular (LV) mass AW, and LV volume as detected by type-B ultrasonic test.

### Echocardiography

The effects of exosomal PFN2 on the cardiac function of MI mouse were evaluated using a high-frequency ultrasound system Vevo2100 (VisualSonics, Canada) equipped with a linear array transducer (MS250, 13–24 MHz). This transducer is specifically designed for mouse, guinea pigs or other similar sizes of small animals. During image acquisition, mice were anesthetized continuously with 2% isoflurane. All animals were placed in the dorsal recumbent position for ultrasound imaging. Ejection fraction (EF) and fractional shortening (FS) were detected by type-B ultrasonic test.

### Transmission Electron Microscopy

For identification of exosomes by TEM, samples were fixed in 0.01 M PBS (pH 7.4) at 4°C overnight. After washing with PBS, the samples were fixed in 1% OsO_4_ for 30 min, rinsed with distilled water, precipitated and dehydrated in concentration gradient alcohol, stained with 1% uranyl acetate for 30 min, and finally embedded in TAAb812. After polymerization at 60°C overnight, the precipitate was sliced, and the ultrathin sections were observed under a Tecnai transmission electron microscope (FEI, USA).

### PFN2 Overexpression and PFN2 Knockdown Endothelial Cells

Human umbilical vein ECs (HUVECs) were cultured in RPMI-1640 medium (SH30809.01, Hyclone, USA). Rat brain microvascular ECs (RBMECs) were cultured in Endothelial Cell Medium (1001, ScienCell, USA) for the first five generations and then cultured in RPMI-1640 medium.

Lentiviral vectors were used for profilin 2 overexpression (PFN2-OE or OE) and profilin 2-knockdown (PFN2-KD or KD) in endothelial cells. The PFN2-OE cells were generated using pLVX-mCMV-ZsGreen-PGK-Puro vector, and PFN2-KD cells were infected with pLVX-shRNA2-Puro. The sequences of profiling 2 primer and profilin 2-shRNA are listed in Supplementary Table 2. HUVECs and RBMECs were transduced for 24 h with recombinant lentivirus and cultured for 72 h. The transduction efficacy was verified by GFP expression as determined by fluorescence microscopy, and subsequently confirmed by qPCR and western blotting.

### Quantitative Polymerase Chain Reaction (QPCR) Analysis

A total of 15 gerbils at different developmental stages [embryo (prenatal), brain, and heart (postnatal)] were used for qPCR analysis (3 samples for each time point) of PFN2 levels during development. The expression levels of ECs were also analyzed with qPCR. qPCR was also performed following the earlier described protocol. Cells were resuspended in medium at a density of 2,000 cells/well. Total RNA was extracted from ECs using TRIzol reagent (Tiangen). We synthesized cDNA using FastQuant RT Kit (Tiangen) following manufacturer's instructions. An iQ5 thermal cycler (Bio-Rad, USA) was used to perform qPCR as follows: pre-denaturation at 95°C for 15 min, 40 cycles of denaturation at 95°C for 10 s, annealing and extension at 60°C for 35 s, and 71 cycles of melt curve analysis at 60°C for 10 s. Primer sequences for profilin 2 and other genes are listed in Supplementary Table 2.

### Vascular Development of Zebrafish

The zebrafish model was established by microinjection of exosomes labeled with the fluorescent carbocyanine dye Dil (red), (Solarbio, D8700-10) into the perivitelline cavity of mp805a zebrafish embryos at 24 h post-fertilization. The embryos were cultured at room temperature, and the distribution of exosomes was observed by fluorescence microscopy from 0 to 48 h after injection. The zebrafish vascular structure and the number of vessels were imaged using a confocal microscope (TCS SP5, Leica).

### Inflammatory Models of the Cell

ECs were treated with 1 μg/mL of LPS for 24 h.

### Real-Time Cell Analyzer (RTCA) Assay

We used a real-time cell analyzer (RTCA) system (ACEA, USA) to explore the effect of PFN2 on endothelial cell migration and proliferation, as described in our previous report (27). Briefly, cells were seeded in E-plates at a density of 1,000 HUVECs/well and 2,000 RBMECs/well. The E-plates were then transferred to the RTCA-Dual Purpose instrument for automated real-time monitoring under standard incubator conditions. Cell index measurements were collected every 5 min. Cellular migration and invasion were also monitored using the RTCA system on cell invasion-and-migration (CIM)-plates instead of E-plates. Cell migration activity was monitored with the impedance readouts. Migration assays were performed by seeding cells in the upper chambers of the CIM-plates in serum-free medium at a density of 10,000 cells/well. The bottom chambers of the CIM-plates were filled with serum-containing medium to promote migration across the membranes along the serum gradient. Data were collected by real-time readouts.

### Enzyme-Linked Immunosorbent Assay (ELISA) and Analysis of Exosomal PFN2

The sera were collected before and after surgery at different time points and PFN2, basic fibroblast growth factor (bFGF), and vascular endothelial growth factor (VEGFA) were detected using ELISA kits. The protein levels in the cell culture supernatant and serum were verified by ELISA. The proteins in the cell culture supernatant were determined using a human PFN2 ELISA kit (Cyagen, USA) and the VEGFA (R&D) kit. The concentrations of PFN2, bFGF, and VEGFA in the serum samples obtained from mouse, patient, pig, and rat with MI were determined using the mouse (Cyagen), human (Cyagen), pig (Cyagen), and rat ELISA kits (Cyagen), respectively. Exosomal PFN2 and VEGFA in the serum were identified using mouse or pig PFN2 and VEGFA kit (Cyagen). All experiments were performed according to the manufacturer's instructions. The level of rat exosomes in the serum was detected by Overall Exosomes Capture and Quantification (Plasma, Colorimetric) kit (NBP2-49782, Novus, USA).

### Endothelial Tube Formation Assay

The *in vitro* effects of PFN2 on HUVEC and RBMEC cell angiogenesis and viability were evaluated by tube formation assay. The tube formation assay was performed using Matrigel^®^ (BD Biosciences), according to the manufacturer's instructions. Matrigel^®^ was thawed overnight at 4°C. The 96-well plate and 100 μl pipette tips were maintained at 4°C overnight. Subsequently, Matrigel^®^ (30 μl/well) was added to the 96-well plate and incubated at 37°C for 1 h. Then, cells were incubated for 24 h at 37°C 5% CO_2_. Tube formation was observed using an inverted phase-contrast microscope. The total branching length of the vascular network was determined using ImageJ.

### MTT Assay

MTT assay as reported previously (27). Briefly, Target cells were resuspended in medium at a density of 2000 cells/well and were allowed to adhere for 6 hours. Wells containing 100-μL medium alone (without cells) were used as negative controls. MTT assays were performed at 48 h. The results for the negative control were used as a baseline. Each experiment was repeated 3 times, and the results are presented as a percentage of viable cells as calculated by the following equation: (mean absorbance of experimental well/mean absorbance of positive control well) × 100 = percentage of viable cells.

### Western Blot Analysis

Protein extraction and western blotting were performed as reported previously (27). The primary antibodies used were as follows: PFN2 (Abcam, ab191054, UK), PI3K (Abcam, ab32089, UK), p-ERK (CST, 4372, USA), VEGFA (Abcam, ab214424, UK), PCNA (Abcam, ab92552, UK), Alix (Abcam, ab186429, UK), and GAPDH (Abcam, ab181602, UK), and were diluted 1:1000. The secondary antibodies were diluted 1:5000. The membranes were washed thoroughly, and protein bands were visualized with enhanced chemiluminescence (ECL) immunoblotting detection reagent (Thermo Fisher Scientific, USA). Semi-quantitative results were normalized to the expression of the housekeeping gene GAPDH, after gray scanning.

### Statistical Analysis

Statistical analysis was performed using SPSS 16.0 (SPSS Inc., USA). After the normality test and variance homogeneity test of measured data, comparisons between different groups were performed using Student's *t*-test, one-way ANOVA, or repeated-measures ANOVA (Tukey). Bar charts show the mean ± SEM. A *p* ≤ 0.05 indicated a significant difference.

## Results

### PFN2 and Exosomal PFN2 Levels Increased in the Serum of Post-MI Patients and the MI Animal Models During Angiogenesis Phase

**Figure 1:**
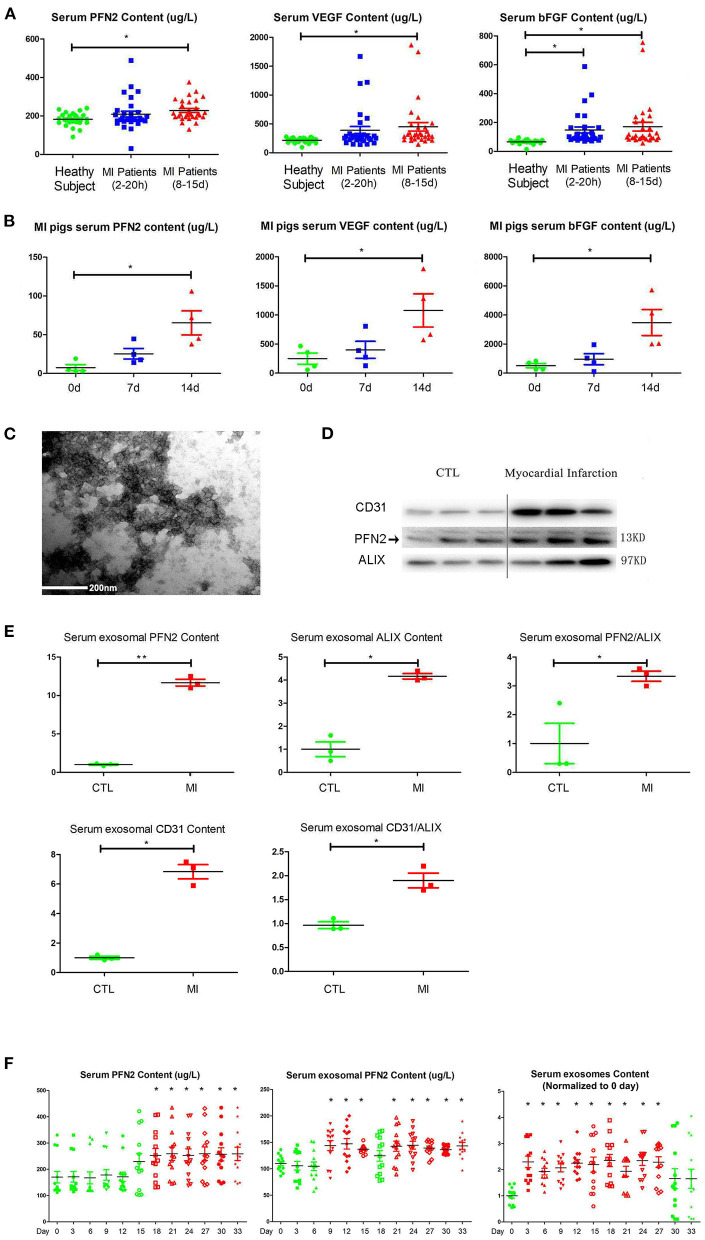
Increased PFN2 and exosomal PFN2 levels in the serum of patients with MI and in animal models. **(A)** Enhanced PFN2 levels, VEGFA and bFGF levels in patients with MI (patients with MI 2–20 h, *n* = 30; MI 8–15 d, *n* = 30; CTL, *n* = 24). **(B)** Enhanced PFN2 levels, VEGFA and bFGF levels in pigs with MI (pigs with MI, *n* = 4; CTL, *n* = 4). **(C)** Identification of serum exosomes in patients with MI by electron microscopy. **(D)** Exosomal PFN2 between CTL (*n* = 3) and MI groups (*n* = 3). **(E)** Exosomal PFN2 levels between CTL (*n* = 3) and MI groups (*n* = 3) (The protein level was normalized to control). **(F)** Serum PFN2 levels, Exosomal PFN2 levels and Exosomes in rats (*n* = 13) before and after MI. *p < 0.05, one-way ANOVA and repeated-measures ANOVA (Tukey).

VEGFA and bFGF are well-known markers for the establishment of collateral circulation after MI (28, 29). We measured the PFN2, VEGFA and bFGF levels in the serum of patients with MI and in pig animal model to explore the relationship between serum PFN2 level and angiogenesis after MI. The results showed increased PFN2 levels in the serum of patients with MI, especially in patients after 8–15 days post-MI (*p* = 0.021) compared to their corresponding CTL, consistent with the levels of VEGFA and bFGF (Figure 1A). We also observed significantly higher levels of PFN2, VEGFA, and bFGF in pigs with MI compared to healthy CTL on days 7 and 14 post-MI, respectively (Figure 1B). These data showed a positive correlation between serum PFN2 levels and markers for the establishment of collateral circulation, which suggested the possible relationship between PFN2 and angiogenesis after MI. Besides, we found that PFN2 was negative associated with creatinine kinase-myocardial band (CK-MB) (Pearson correlation = −0.321, *p* = 0.012) and diastolic blood pressure (DBP) (Pearson correlation = −0.300, *p* = 0.020) (Supplementary Figure 1), which both indicated poor prognosis of MI (30, 31).

To examine if PFN2 was delivered intracellularly by exosomes in serum, we isolated exosomes from the serum of patients with MI and, after identification by TEM, tested the exosomal PFN2 level between the CTL and MI groups. The data indicated that exosomes were present in the MI serum (Figure 1C). In addition, the exosomal CD31 and PFN2 levels in the MI group were significantly higher than those in the CTL group (*p* = 0.035/*p* = 0.025) (Figures 1D,E), which suggested that elevated PFN2 content might be associated with endothelial cells derived exosomes increasing in MI patients serum.

We established a rat model of MI by coronary artery ligation and then analyzed the dynamic changes in serum PFN2, exosomes, and exosomal PFN2 at different time points after surgery. Intriguingly, serum exosomes, serum exosomal PFN2 levels, and serum PFN2 levels increased significantly after MI on the 3rd day (*p* = 0.037), 9th day (*p* = 0.028), and 18th day (*p* = 0.043), respectively (Figure 1F). These data implicate the association of PFN2 as well as exosomal PFN2 with MI.

### Exosomal PFN2 Reduces Infarction in Post-MI Mouse Hearts and Enhances Exercise Endurance Capacity in Post-MI Mice

**Figure 2:**
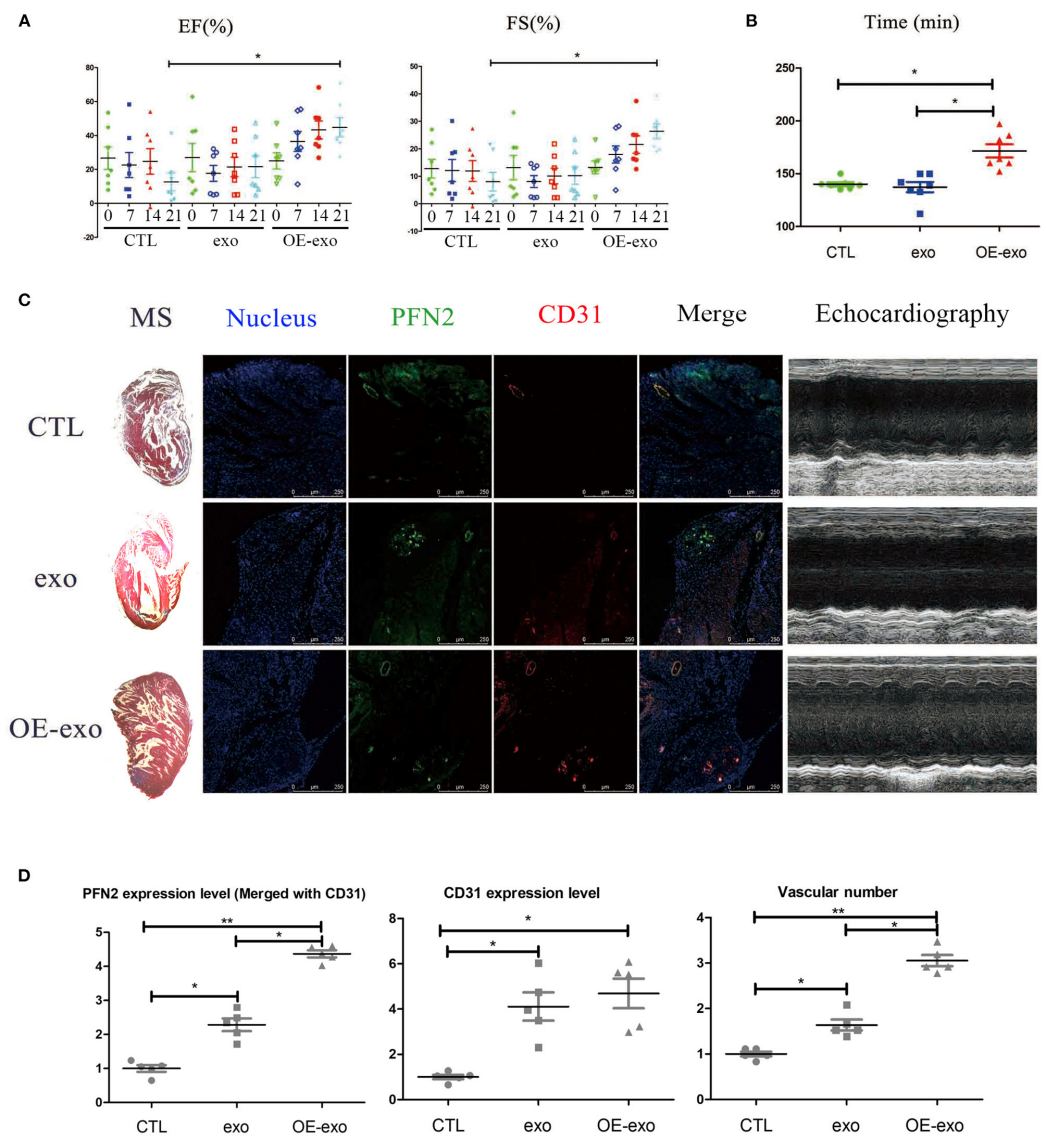
Exosomal PFN2 repairs infarction in mouse with MI and restores cardiac function. **(A)** The cardiac function of mouse with MI as evaluated by type-B ultrasonic test EF and FS after treatment with exosomes demonstrated the benefit to MI impairment. **(B)** Physical fitness was evaluated by fatigue test after treatment with exosomes in mouse with MI which displayed significant improvement of heart function, *n* = 7. **(C)** Infarction volume (Masson's staining) decreased and PFN2 (green)/CD31 (red) staining increased in heart tissue of mouse with MI after treatment with exosomes. **(D)** Statistical results of **(C)**. *n* = 3, normalized to control. *p < 0.05, one-way ANOVA (Tukey).

We tested the cardiac function and repair of infarction in mice with MI 30 days after injecting exo, OE-exo, and saline by physical fitness tests and echocardiography. The results showed significantly improved EF and FS (two critical indicators reflecting cardiac function) in the OE-exo-injected group, higher than the exo and saline groups (Figure 2A). The running time in exo- and saline-injected mice with MI were lower than in OE-exo-injected mice (Figure 2B). These data indicated that OE-exo injection could improve global cardiac function. Masson's staining, echocardiography and Immunofluorescence staining results showed decreased infarction volume and increased vasculature as well as PFN2 (merged with CD31) levels in the heart vessels of OE-exo-injected mice with MI (Figures 2C,D). Together, these results demonstrate that exosomal PFN2 treatment effectively promotes the recovery of cardiac function.

### Exosomal PFN2 Protects Endothelial Cells Against Inflammation

**Figure 3:**
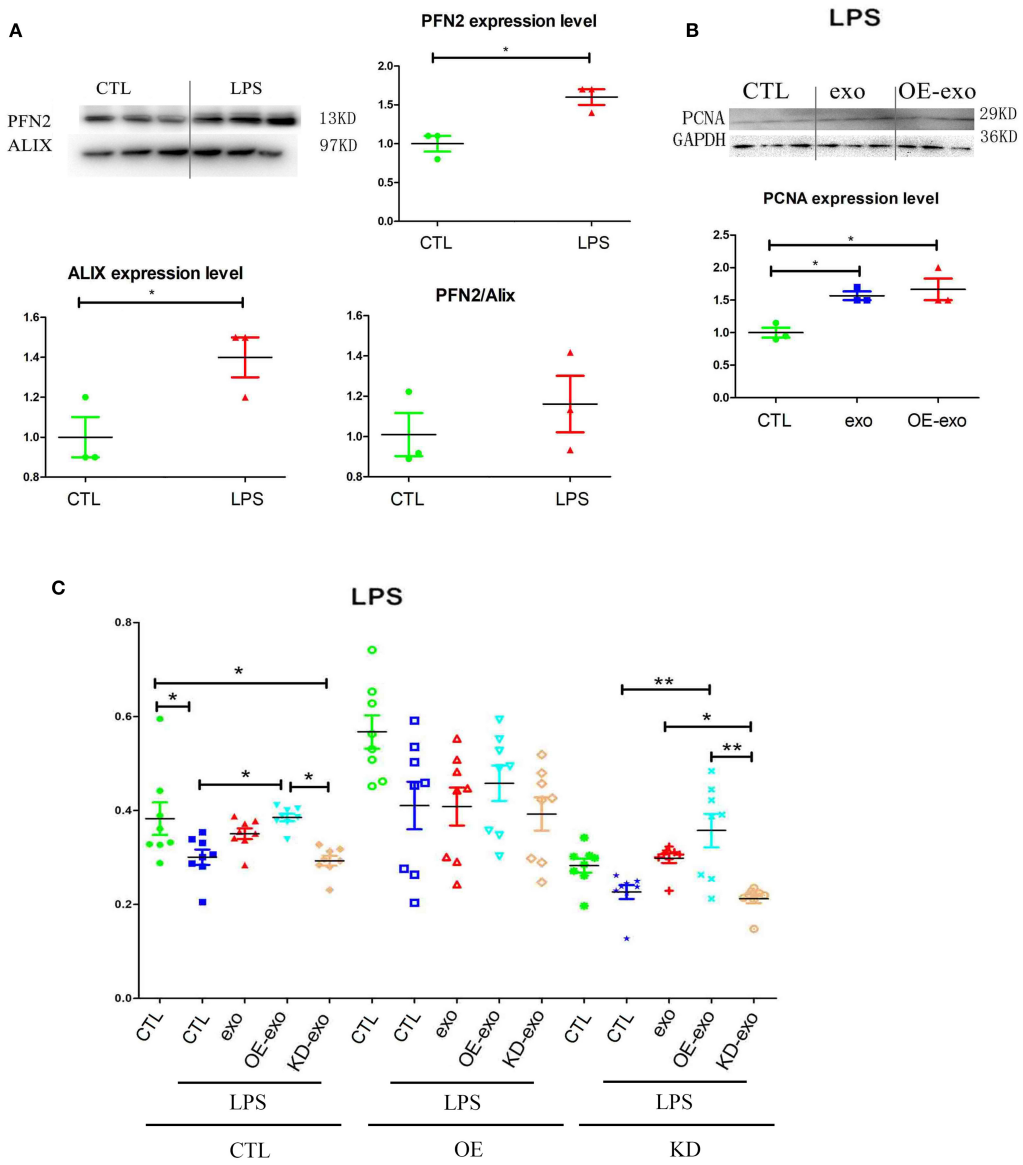
PFN2 protects ECs after LPS treatment. **(A)** PFN2 and Alix expression levels in LPS-treated EC exosomes, *n* = 3, normalized to control. **(B)** PCNA expression levels of ECs under LPS stimulus after treatment with various exosomes, *n* = 3, normalized to control. **(C)** Viability of ECs under LPS stimulation after treatment with various exosomes. *n* = 8. *p < 0.05, one-way ANOVA (Tukey).

Since PFN2 and exosomal PFN2 enhance the proliferation and migration of ECs, we hypothesized that PFN2 might protect ECs from inflammatory stimulus. First, our results showed that LPS stimulation increased the levels of both PFN2 and the exosomes marker Alix, indicating elevated exosomes secretion by ECs after LPS treatment (Figure 3A).

When HUVECs were treated with exo, OE-exo, or KD-exo under LPS stimulus, proliferation of the cells increased in the OE-exo group compared to that in the exo group (Figures 3B,C). Compared to normal cells, LPS could block the proliferation of HUVECs (Figure 3B), but this blocking effect could be reduced by exo, especially the exosomes from OE cells, while KD-exo had limited effects. Overexpression of PFN2 could partially prevent the blocking of proliferation induced by LPS treatment in ECs (Figure 3B). Taken together, our results suggest that exosomal PFN2 could increase the viability of ECs after LPS treatment.

### Exosomal PFN2 Increases Angiogenic Ability *in vitro* and *in vivo*

To determine whether exosomal PFN2 promotes angiogenesis, we first overexpressed the PFN2 in the endothelial cells (Supplementary Figure 2) followed by isolation of the exosomes. We labeled OE-exo with Dil (red) and co-cultured them with HUVECs to explore if exosomes could be internalized by ECs. Next, we treated the endothelial cells with the exosomes carrying overexpressed PFN2.

**Figure 4:**
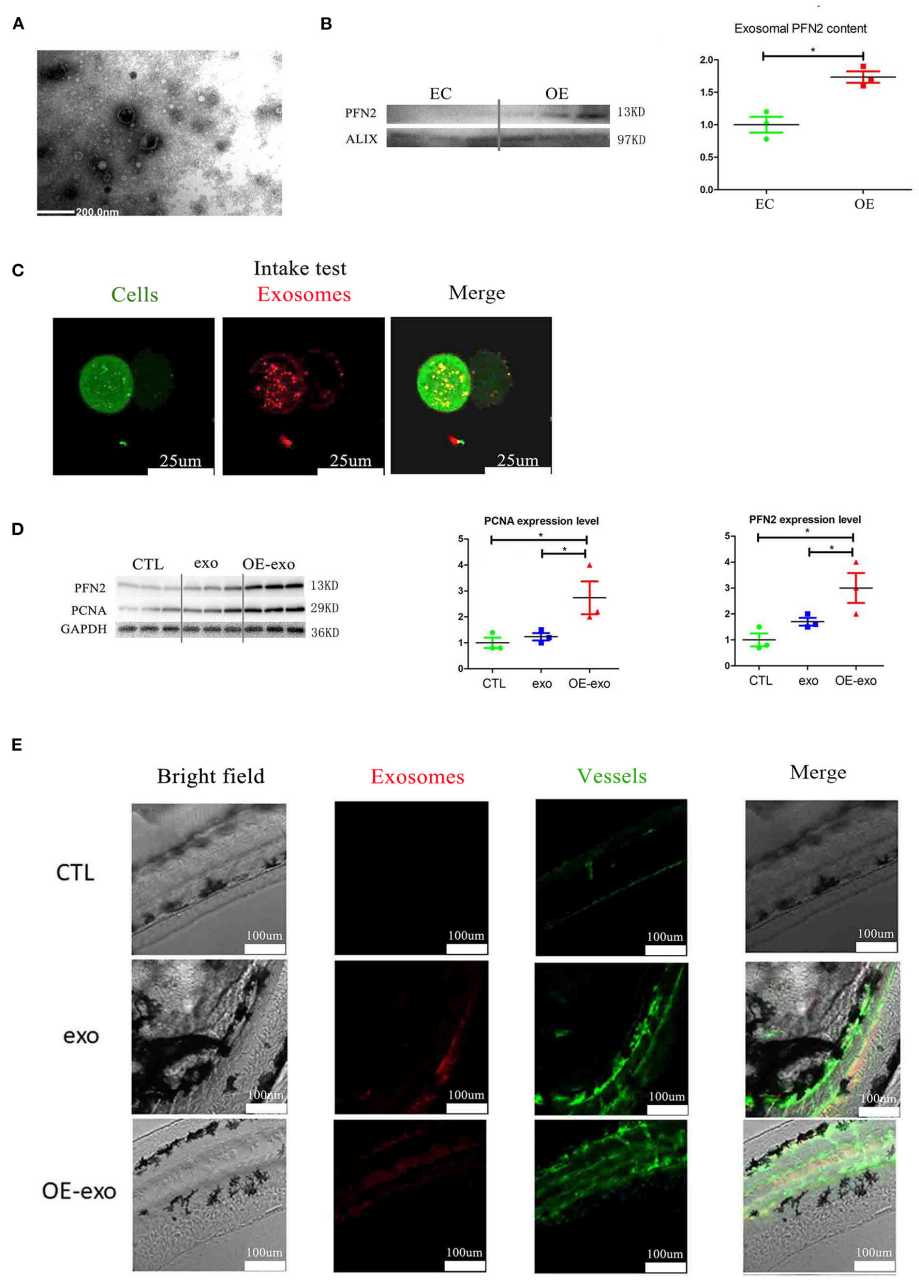
Exosomal PFN2 enhances the proliferation ability of ECs and OE-exo treated zebrafish showed significantly increased vessel number than exo and control. **(A)** The exosomes secreted from ECs were identified by electron microscopy. **(B)** PFN2 expression in exosomes (*n* = 3, the expression levels were normalized to control). **(C)** Exosomes could be internalized by ECs. **(D)** PFN2 and PCNA expression levels in CTL, exosomes from normal ECs (exo), PFN2-overexpressing ECs (OE-exo), and PFN2 knockdown ECs (KD-exo), *n* = 3, and the expression levels were normalized to control. **(E)** OE-exo-treated zebrafish showed significantly increased vessel number than exo and control. Exo, exosomes from normal ECs; OE-exo, exosomes from PFN2-overexpressing ECs; CTL, PBS as control. *p < 0.05, one-way ANOVA (Tukey).

We isolated exosomes from EC culture medium and identified them by TEM and western blotting. We detected the secretion of PFN2-containing exosomes by ECs (Figures 4A,B); the exosomes secreted by OE-ECs had significantly higher PFN2 protein levels than the control ECs (Figure 4B) (*p* = 0.042). And the results showed internalization of OE-exo into the perinuclear compartment in HUVECs (Figure 4C). Then the effect of exosomes isolated from normal ECs (exo), and PFN2-overexpressing ECs (OE-exo) on the proliferation of ECs was investigated. The results showed that the level of PCNA increased in ECs in the OE-exo group (*p* = 0.048) compared to the exo group (Figure 4D).

However, when ECs were directly treated with PFN2 protein, it could not increase the proliferation and migration of ECs (Supplementary Figure 3), implying that exosomes are crucial for PFN2-induced angiogenic ability in ECs.

Larval zebrafish are commonly used in vascular research because they are transparent, and their vessels can be visualized directly. We explored the effect of OE-exo on angiogenesis in zebrafish. The results showed increased angiogenesis in larval zebrafish after treatment with exosomes, and OE-exo showed the highest effect (Figure 4E). These data suggest that transportation of PFN2 by exosomes increases HUVEC proliferation and migration as well as angiogenesis.

### Elevation of PFN2 Is Associated With Vascular Development and Promotes the Tube Formation

**Figure 5:**
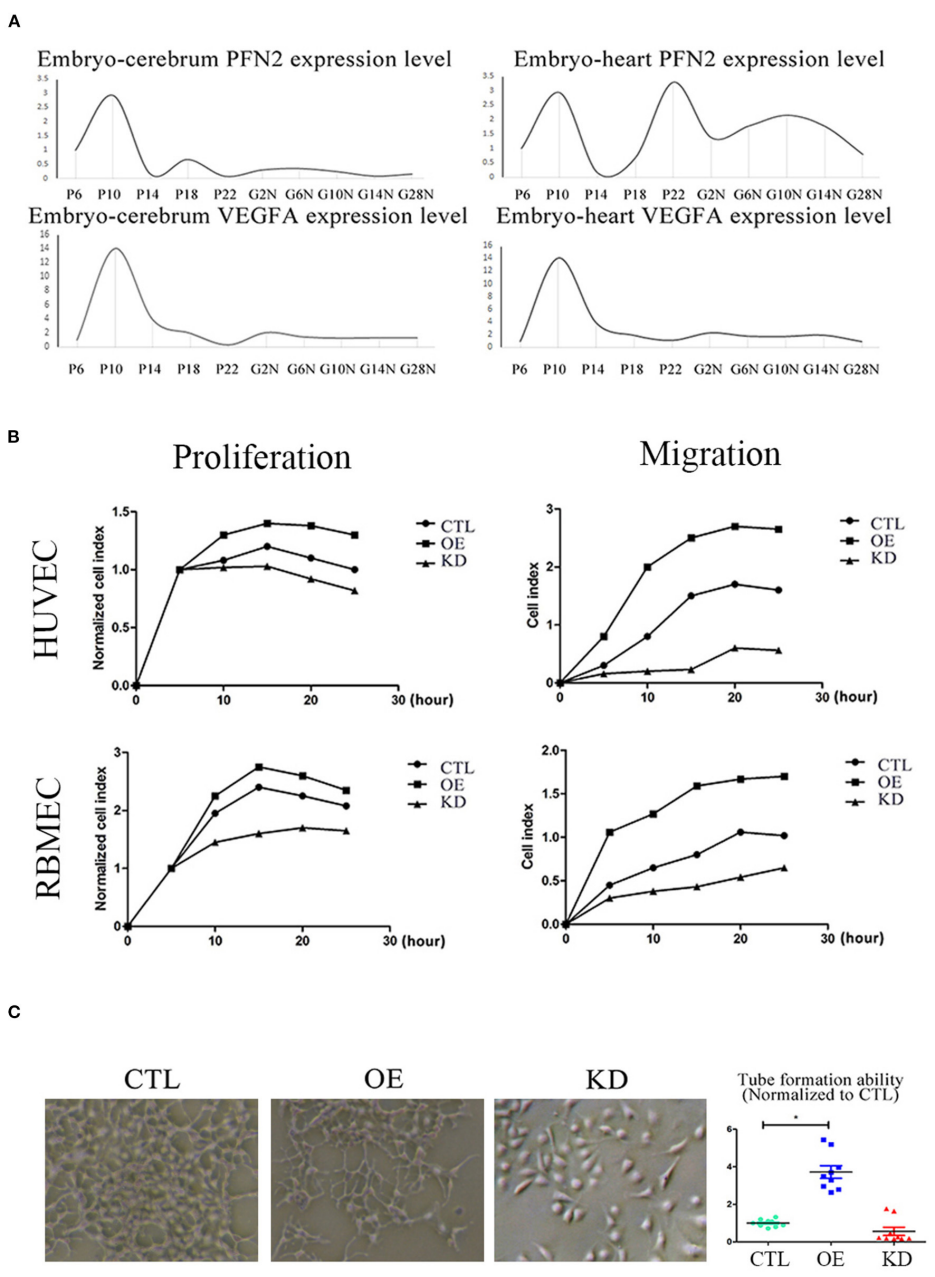
PFN2 promotes proliferation and migration of ECs, as well as tube formation and reaches the maximum level in 10-day embryo. **(A)** The mRNA levels of PFN2 and VEGFA at different developmental stages of gerbils (*n* = 15) embryo (prenatal), brain and heart (postnatal). **(B)** PFN2 significantly promoted proliferation of HUVECs (*n* = 3) and RBMECs (*n* = 3), as well as showed a positive effect on migration of HUVECs (*n* = 3) and RBMECs (*n* = 3). **(C)** The tube length in PFN2-overexpressing ECs (OE) was significantly increased than that in blank CTL and PFN2 knockdown ECs (KD), *n* = 3. *p < 0.05, one-way ANOVA and repeated-measures ANOVA (Tukey).

To understand the relationship between PFN2 and angiogenesis, we explored the role of PFN2 in gerbil embryo development using VEGFA as a positive control. The results showed that both PFN2 and VEGFA reached maximal expression levels on day 10 of the embryo stage (Figure 5A). Since day 10 (embryo) is a critical time point for vascular development, this result shows the important association of PFN2 with vascular formation.

To investigate the role of PFN2 in angiogenesis and its influence on the proliferation and migration of both venous and arterial ECs, we established PFN2 overexpression (OE) and knockdown (KD) EC lines using HUVECs (venous ECs) and RBMECs (arterial ECs). PFN2 RNA expression (Supplementary Figure 2A) and protein levels (Supplementary Figure 2B) increased significantly in OE cells, with a significant decrease in KD cells. We performed experiments using PFN2-OE and PFN2-KD in both HUVECs and RBMECs by RTCA analysis (Figure 5B). The results showed that OE-HUVECs exhibited greater proliferation and migration ability (*p* = 0.044; *p* = 0.035), while KD-HUVECs showed reduced proliferation (*p* = 0.037) and migration (*p* = 0.030). Similar results were observed in OE-RBMECs (*p* = 0.040; *p* = 0.038) as well as KD-RBMECs (*p* = 0.040 and *p* = 0.029, respectively). The results of the tube formation test demonstrated that compared to the control and KD-ECs, OE-ECs showed a significantly increased tube length (*p* = 0.038) (Figure 5C). These results indicate that PFN2 could significantly increase the proliferation, migration and the angiogenic ability of ECs.

### PFN2 Regulates Angiogenesis via the PI3K and ERK Signaling Pathways

**Figure 6:**
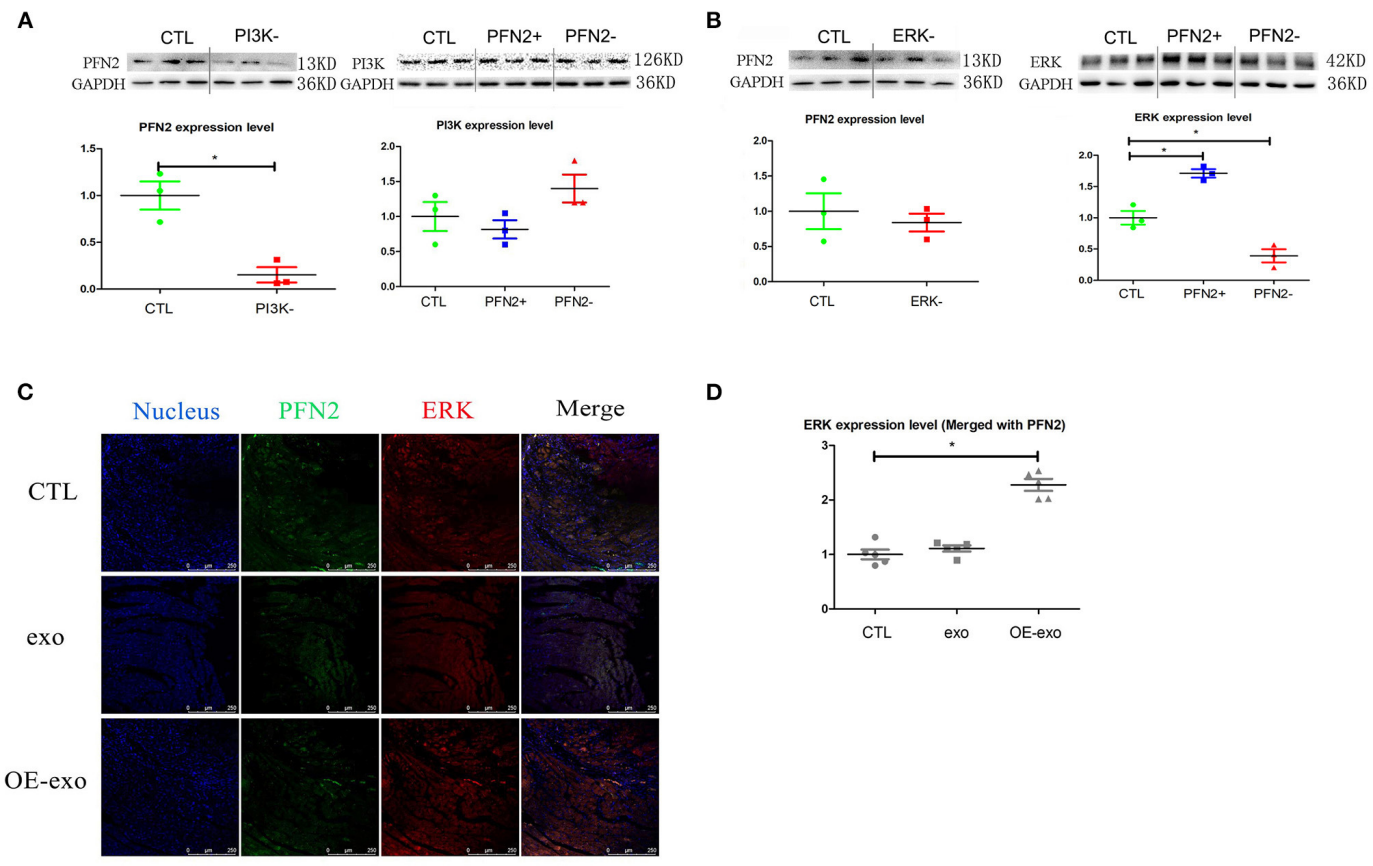
PFN2 involved molecular signaling pathway. **(A)** PFN2 expression level in HUVECs treated with PI3K inhibitor and PI3K expression level in OE and KD, *n* = 3, normalized to control. **(B)** PFN2 expression level in HUVECs treated with ERK inhibitor and ERK expression level in OE and KD, *n* = 3, normalized to control. **(C)** ERK and PFN2 expression levels in the heart tissue of MI mice treated with different exosomes, *n* = 3, normalized to control. **(D)** Statistical result of **(C)**. *p < 0.05, Student's *t*-test and one-way ANOVA (Tukey).

We further explored the potential signaling pathways through which PFN2 was involved in angiogenesis. We identified the regulatory relationship between angiogenesis-related proteins and PFN2. When PI3K was inhibited, PFN2 expression level was reduced (*p* = 0.024), while PFN2 overexpression increased the expression of ERK (*p* = 0.025) and its knockdown decreased ERK expression (*p* = 0.031) (Figures 6A,B). However, VEGFA had no influence on PFN2 levels in HUVECs. Similarly, PFN2 overexpression or knockdown also had no effect on VEGFA levels in ECs (Supplementary Figure 4).

Importantly, we found ERK (merged with PFN2) levels were increased in the post-MI murine hearts treated with OE-exo (Figures 6C,D).

These data demonstrate that PFN2 promotes angiogenesis through the PI3K-PFN2-ERK axis, and is not associated with VEGFA. Moreover, we performed a rescue experiment to determine whether the PI3K-PFN2-ERK axis contributed to EC angiogenesis. PI3K or ERK inhibition in OE-EC cells was mimicked by LY294002 or HY-50846, respectively. These data further confirm that PFN2 enhances EC angiogenesis through the PI3K-PFN2-ERK axis (Graphical Abstract).

## Discussion

The present study, for the first time, demonstrates an increased level of serum PFN2 and exosomal PFN2 in patients and animals with MI, which positively correlated with bFGF and VEGFA levels. PFN2 and exosomal PFN2 impact the angiogenic ability of ECs *in vitro*, and exosomal PFN2 treatment significantly increased the vessel number in zebrafish *in vivo*. More importantly, endothelial exosomal PFN2 dramatically improved cardiac function and physical motion in mice with MI, thereby repairing MI injury. It is known that current clinical treatments for MI aim to alleviate symptoms by surgical interventions and by dissolving arterial blockage by injection of thrombolytic or clot-dissolving drugs. However, due to the patient's clinical condition or limitation of available technology, a significant number of MI cases would be ineligible to receive therapy either by surgical interventions or by clearance of arterial blockage (32). As a result, the development of advanced strategies based on improving angiogenesis in some cases is critical to prevent or decrease MI injury (33, 34). Our results which focus on enhancing the proliferative and migratory ability of ECs by endothelial exosomal PFN2 may be a promising strategy for therapeutic angiogenesis treatment for MI. Since endothelial exosomes are relatively safe, our current strategy is feasible and efficient for future clinical treatment of MI.

PFN2 is a member of the profilin family and shares 80% similarity with PFN1. We selected PFN2 due to its association with variations in the circle of Willis (35). An earlier report described that PFN2 is specifically expressed in neuronal cells and kidney cells (36). Unlike reports on other profilin family members such as PFN1, studies on PFN2 are quite few and are mainly related to tumors (37, 38). Studies on the role of PFN2 in angiogenesis are rare. Very recently, we found that exosomal PFN2 from small cell lung cancer (SCLC) cells could promote the growth of these cells, as well as significantly improve the proliferation, migration, and tube formation of ECs (37). Furthermore, we found that PFN2 reached maximum levels in 10-day gerbil embryos, which is the critical vascular development time point in animals. To explore the effect of PFN2 on angiogenesis, we showed that overexpression of PFN2 enhanced the proliferative, migratory, and tube formation abilities of ECs. Moreover, endothelial exosomal PFN2 ameliorated cardiac function after MI. The effect of PFN2 on angiogenesis explored in the current study is similar to PFN1, which is expected to be a new target for the treatment of MI as reported in a recent study (39). Our current results partly corroborate the findings in this report, but more adequately confirmed that exosomal PFN2 could improve angiogenesis and thereby restore cardiac function. We showed that the PFN2-enriched exosomes from ECs increased the number of vessels in zebrafish, a compelling evidence that PFN2 may be regarded as a novel angiogenic molecule. Moreover, our study revealed that serum PFN2 levels increased in patients, rats, mice, and pigs, with MI accompanied with increased levels of VEGFA and bFGF, well-known growth factors for angiogenesis (28, 29), suggesting that PFN2 might be an effective complementary marker for collateral vessel development in MI. We monitored the dynamic changes in PFN2, exosomes, and exosomal PFN2 after coronary artery occlusion, all of which increased in the serum of rats with MI and reached maximal exosomes numbers earlier than the exosomal PFN2, and PFN2. Thus, we established 7-days post-MI as the time point for injection of OE-exo for maximum exosomal PFN2 expression. The above strategy using OE-exo resulted in a comparable, suitable and efficient therapy for mice with MI.

Several molecular pathways are involved in angiogenesis during MI repair (40, 41). VEGFA is the most common contributor to angiogenesis and manifests cardioprotective effects in the repair of infarcted heart (40, 42). Therefore, we explored whether the angiogenic function of PFN2 was via the VEGFA pathway. However, our results illustrated that overexpression or knockdown of PFN2 in ECs had no impact on the level of VEGFA, and vice versa (Supplementary Figure 3). These results showed that PFN2 was involved in angiogenesis independent of the VEGFA pathway in ECs. However, these findings are different from that reported by Tang YN et al. where they observed that PFN2 promoted tumor growth of non-small cell lung cancer by PFN2-SMAD2/3-VEGFA pathway (36). Further, our investigation regarding the molecular mechanism indicated that the PI3K-PFN2-ERK axis is involved in PFN2-mediated angiogenesis. These data are consistent with our work in SCLC which showed that exosomal PFN2 from SCLC cells activated Smad2/3 in these cells and pERK in ECs. Interestingly, Hao P et al. reported significantly higher PFN1 levels in the heart tissues of rats with MI than in control animals, and together with pERK levels positively correlated with the levels of CK-MB, EMP, and others (43). Our present results preliminarily confirm that PFN2 could enhance ERK levels as well as suggests a novel mechanism that affects EC angiogenesis by the PFN2-ERK axis.

As an emerging therapeutic candidate for MI treatment, exosomes have been described to mediate local and distant micro-communication between cardiomyocytes and endothelial cells (44). Todorova et al. reported that exosomes were involved in EC proliferation, migration, sprouting, branching, and tube formation (17). Accumulating literature has proved that the exosomes involved in the repair of MI, as an angiogenic mediator, could be secreted from bone marrow cells (45), cardiomyocytes (46), and endothelial progenitor cells (47). Exosome cargos vary from miRNAs (48), LncRNAs (49), and proteins (19). It has been reported that cardiomyocytes and endothelial cells could interact or communicate with exosomes and promote angiogenesis in ECs (50). Furthermore, targeting endothelial exosomes is beneficial for the prevention of cardiovascular diseases (51). Considering that PFN2 is an intracellular protein, we hypothesized that it may need a medium to be delivered from donor cells to recipient cells. As we expected, PFN2-enriched exosomes (OE-exo) derived from ECs could promote angiogenesis *in vivo* and *in vitro*, repair EC injury under inflammatory stimulus, and significantly attenuate MI injury. However, in contrast to exosomal PFN2, PFN2 protein could not directly increase, even slightly, the viability and migration of ECs (Supplementary Figure 3), implying that exosomes are critical mediators for PFN2-mediated angiogenic ability. Recently studies have provided insights into the exosomal cancer-homing behavior of different tumor cell lines, indicating that exosomes have the ability to target their parental cells (52). In the current study, the exosomes we focused on were from the normal human endothelial cell line, which had a higher target efficiency in angiogenesis than other types of cells. Song et al. reported that extracellular vesicles loaded with miRNA-21 isolated from HEK293T cells could effectively inhibit apoptosis of cardiomyocytes and ECs, therefore used for rescue of MI, showing no inflammation with 293T-exosomes (53). Our endothelial cell-derived exosomes might have more biological safety. Moreover, OE-exo from the EC line is available more readily and has less immunological risk than exosomes from explant-derived cardiac stromal cells from heart failure patients and normal donor hearts (54). Taken together, this study provides a new insight into the role of exosomes and for the first time confirms the potential use of endothelial OE-PFN2-exo in treating myocardial infarction (Graphical Abstract).

## Conclusions

In summary, PFN2 and endothelial exosomal PFN2 promoted EC angiogenic ability and protected ECs from inflammation, consequently alleviating infarction and enhancing cardiac function in MI, mediated by the PI3K-PFN2-ERK axis. This study, for the first time, showed that PFN2 plays a role in angiogenesis and that endothelial exosomal PFN2 could be a prospective therapeutic strategy for treating MI.

## Data Availability Statement

The raw data supporting the conclusions of this article will be made available by the authors, without undue reservation.

## Ethics Statement

The studies involving human participants were reviewed and approved by Ethics Committee of Anzhen Hospital. The patients/participants provided their written informed consent to participate in this study. The animal study was reviewed and approved by Animal Experiments and Experimental Animal Welfare Committee of Capital Medical University.

## Author Contributions

ZL, XH, ZC, and XD designed the research studies and wrote the manuscript. ZL, XH, KC, FY, WT, QZ, HY, HC, BZ, and YW conducted the experiments. ZL, XH, KC, and HC acquired the data. ZL and XH analyzed the data. ZL, XH, QZ, HY, and YW collected clinical samples and information. All authors contributed to the article and approved the submitted version.

## Funding

This study was funded by the National Natural Science Foundation of China (Nos. 32070531, 31970512).

## Conflict of Interest

The authors declare that the research was conducted in the absence of any commercial or financial relationships that could be construed as a potential conflict of interest.

## Publisher's Note

All claims expressed in this article are solely those of the authors and do not necessarily represent those of their affiliated organizations, or those of the publisher, the editors and the reviewers. Any product that may be evaluated in this article, or claim that may be made by its manufacturer, is not guaranteed or endorsed by the publisher.
