# Supplementary material for: Profilin 2 and Endothelial Exosomal Profilin 2 Promote Angiogenesis and Myocardial Infarction Repair in Mice
Source: Front Cardiovasc Med. 2022 Apr 11;9:781753. doi: 10.3389/fcvm.2022.781753 (PMC9036097; doi:10.3389/fcvm.2022.781753)
Supplement: Supplementary file 1 [file Data_Sheet_1.docx]

**Table S1 Information of MI patients**

| Number | Group | Diagnose | Gender:  male 1, female 0 | Age | Time of onset of illness |
| --- | --- | --- | --- | --- | --- |
| 01 | 2 | MI | 0 | 66 | 10d |
| 02 | 2 | MI | 0 | 49 | 14d |
| 03 | 2 | MI | 0 | 61 | 14d |
| 04 | 2 | MI | 0 | 65 | 8d |
| 05 | 2 | MI | 0 | 76 | 9d |
| 06 | 2 | MI | 0 | 76 | 14d |
| 07 | 2 | MI | 0 | 52 | 13d |
| 08 | 2 | MI | 0 | 79 | 10d |
| 09 | 2 | MI | 0 | 63 | 10d |
| 10 | 2 | MI | 1 | 41 | 10d |
| 11 | 2 | MI | 0 | 76 | 10d |
| 12 | 2 | MI | 0 | 72 | 14d |
| 13 | 2 | MI | 0 | 72 | 15d |
| 14 | 1 | MI | 0 | 66 | 4h |
| 15 | 2 | MI | 1 | 69 | 10d |
| 16 | 1 | MI | 0 | 61 | 6h |
| 17 | 1 | MI | 0 | 65 | 9h |
| 18 | 2 | MI | 1 | 53 | 10d |
| 19 | 1 | MI | 0 | 61 | 7h |
| 20 | 1 | MI | 0 | 47 | 10h |
| 21 | 2 | MI | 0 | 78 | 11d |
| 22 | 1 | MI | 0 | 75 | 7h |
| 23 | 1 | MI | 0 | 61 | 5h |
| 24 | 1 | MI | 0 | 73 | 5h |
| 25 | 1 | MI | 0 | 51 | 12h |
| 26 | 1 | MI | 0 | 70 | 5h |
| 27 | 2 | MI | 0 | 69 | 14d |
| 28 | 2 | MI | 1 | 62 | 10d |
| 29 | 2 | MI | 1 | 42 | 13d |
| 30 | 2 | MI | 1 | 66 | 13d |
| 31 | 2 | MI | 1 | 62 | 10d |
| 32 | 1 | MI | 0 | 67 | 2h |
| 33 | 2 | MI | 1 | 52 | 10d |
| 34 | 1 | MI | 0 | 68 | 4h |
| 35 | 2 | MI | 1 | 40 | 11d |
| 36 | 2 | MI | 1 | 56 | 8d |
| 37 | 2 | MI | 1 | 53 | 8d |
| 38 | 2 | MI | 1 | 56 | 10d |
| 39 | 1 | MI | 1 | 35 | 7h |
| 40 | 1 | MI | 1 | 50 | 9h |
| 41 | 2 | MI | 0 | 77 | 10d |
| 42 | 1 | MI | 1 | 48 | 4h |
| 43 | 1 | MI | 1 | 58 | 3h |
| 44 | 1 | MI | 1 | 47 | 3h |
| 45 | 1 | MI | 1 | 44 | 5h |
| 46 | 1 | MI | 1 | 42 | 19h |
| 47 | 1 | MI | 0 | 64 | 2h |
| 48 | 1 | MI | 1 | 64 | 10h |
| 49 | 1 | MI | 1 | 69 | 10h |
| 50 | 1 | MI | 1 | 68 | 5.5h |
| 51 | 1 | MI | 1 | 61 | 15h |
| 52 | 2 | MI | 1 | 67 | 10d |
| 53 | 1 | MI | 1 | 42 | 20h |
| 54 | 2 | MI | 1 | 40 | 10d |
| 55 | 1 | MI | 0 | 67 | 8h |
| 56 | 1 | MI | 0 | 58 | 2h |
| 57 | 1 | MI | 1 | 48 | 12h |
| 58 | 1 | MI | 1 | 47 | 4h |
| 59 | 2 | MI | 1 | 61 | 10d |
| 60 | 1 | MI | 1 | 45 | 6h |

**Table S2 The sequences of shRNA and PFN2**

|  | Sequences |
| --- | --- |
| hPFN2 Cloning  primers | pLVX-hPFN2-EcoRI-F：423bp  CGGAATTCGCCACCATGGCCGGTTGGCAGAGCTACGTGG  pLVX-hPFN2-BamHI-R：  CGGGATCCTTACACATCAGACCTCCTCAGGTAT |
| shRNA-primers | shPFN2-F：GATCCGCAGAGCTACGTGGATAACCTTTCAAGAGAAGGTTATCCACGTAGCTCTGCTTTTTTCTCGAGG  shPFN2-R：AATTCCTCGAGAAAAAAGCAGAGCTACGTGGATAACCTTCTCTTGAAAGGTTATCCACGTAGCTCTGCG |
| Primers | β-actin F: AGAGGGAAATCGTGCGTGAC  β-actin R: CAATAGTGATGACCTGGCCGT |
| Primers | PFN2 F: AGTGCGAAGGGCTCGAAGATG  PFN2 R: CTTACACATCAGACCTCCTCAGG |


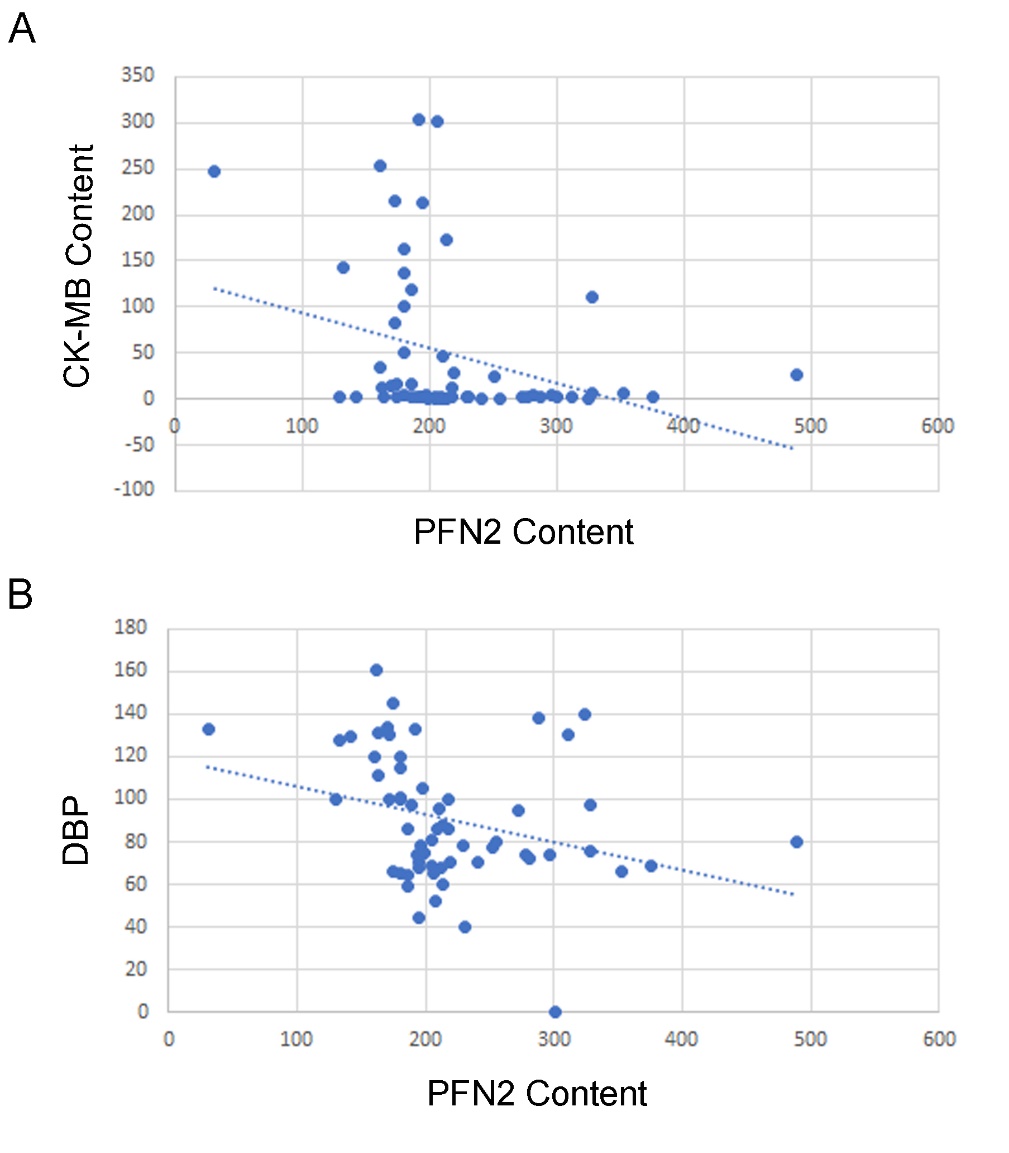


**Fig. S1. Correlation between serum PFN2 content and CK-MB/ DBP.** (A) Correlation between serum PFN2 content and CK-MB; (B) Correlation between serum PFN2 content and CK-MB/ DBP. CK-MB: Creatinine kinase-myocardial band; DBP: diastolic blood pressure.


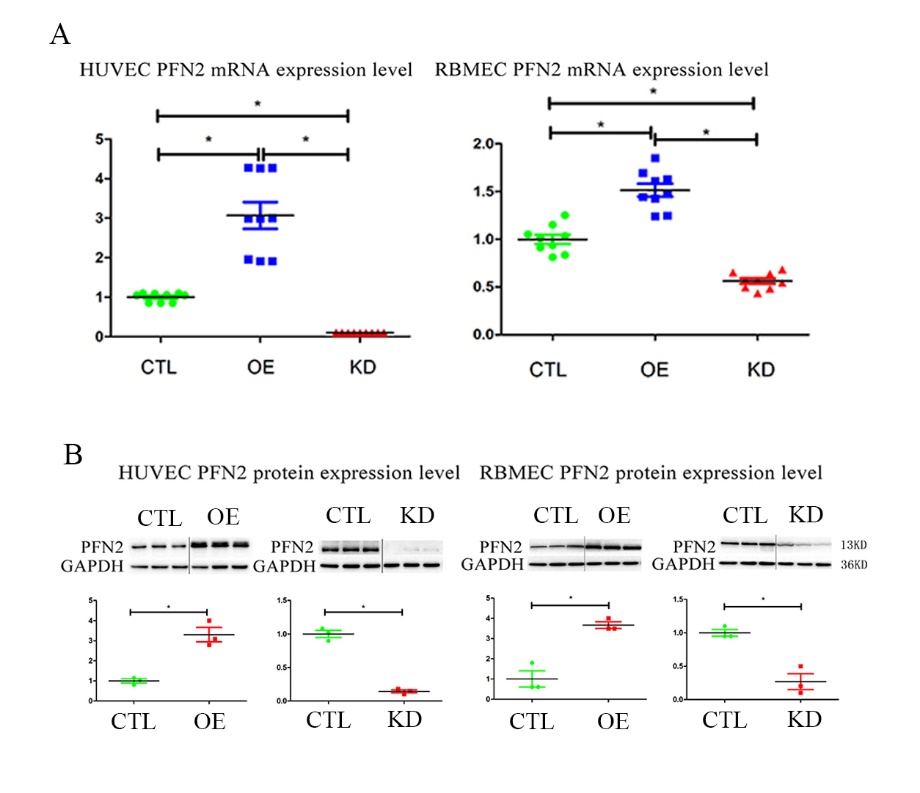


**Fig. S2. Identification of PFN2 overexpression and knockdown ECs.** (A) PFN2 expression level in OE and KD as detected by qPCR (n=9), and the expression levels were normalized to control. (B) PFN2 expression level in OE and KD as detected by western blotting (n=3), and the expression levels were normalized to control. *: *p*<0.05, one-way ANOVA and repeated-measures ANOVA (Tukey).


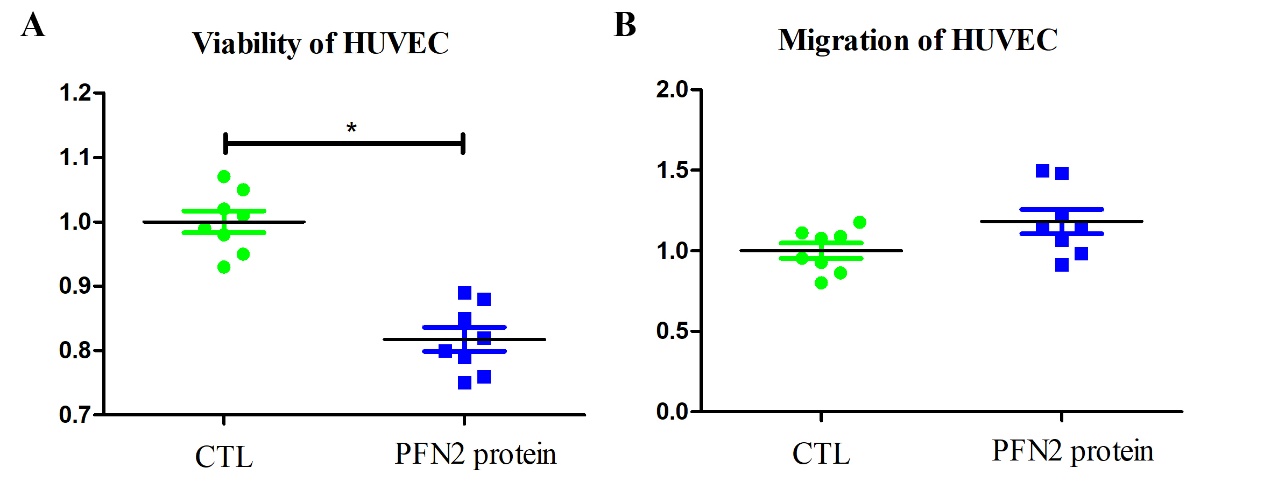
**Fig. S3. Effects of PFN2 protein on EC viability and migration.** (A) Effects of PFN2 protein on EC viability and migration. (B) Effects of PFN2 protein on EC viability and migration. *: *p*<0.05.

**
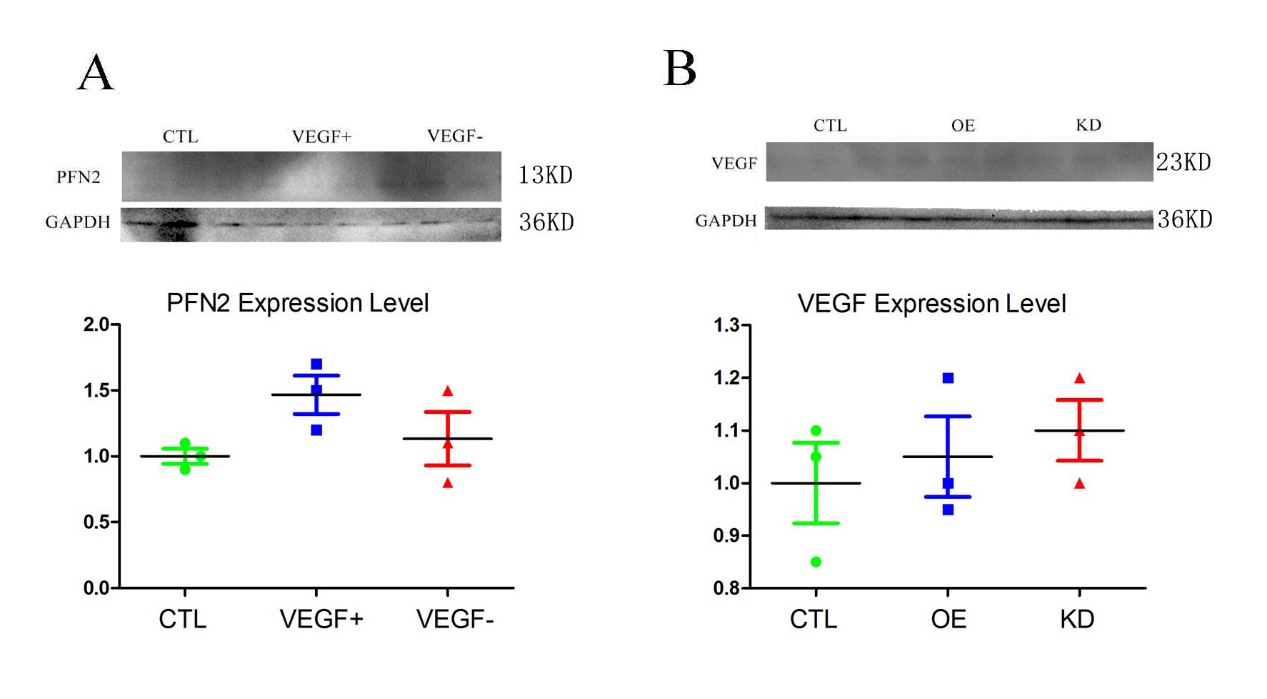
**

**Fig. S4. Interaction effects between PFN2 and VEGF.** (A) Effects of VEGF protein and blocking peptide on PFN2 expression levels. (B) Effects of PFN2 overexpression and knockdown on VEGF expression levels.
